# γδ T cells compose a developmentally regulated intrauterine population and protect against vaginal candidiasis

**DOI:** 10.1038/s41385-020-0305-7

**Published:** 2020-05-29

**Authors:** L. Monin, D. S. Ushakov, H. Arnesen, N. Bah, A. Jandke, M. Muñoz-Ruiz, J. Carvalho, S. Joseph, B. C. Almeida, M. J. Green, E. Nye, S. Hatano, Y. Yoshikai, M. Curtis, H. Carlsen, U. Steinhoff, P. Boysen, A. Hayday

**Affiliations:** 1 ImmunoSurveillance Lab, The Francis Crick Institute, London, NW1 1AT UK; 2 Peter Gorer Department of Immunobiology, School of Immunology and Microbial Sciences, King’s College London, London, SE1 9RT UK; 3 Faculty of Veterinary Medicine, Norwegian University of Life Sciences (NMBU), N-0102 Oslo, Norway; 4 Bioinformatics and Biostatistics Team, The Francis Crick Institute, London, NW1 1AT UK; 5 Experimental Histopathology Laboratory, The Francis Crick Institute, London, NW1 1AT UK; 6 Centre for Host-Microbiome Interactions, Faculty of Dentistry, Oral & Craniofacial Sciences, Guy’s Hospital, King’s College London, London, SE1 9RT UK; 7 Division of Immunology and Genome Biology, Medical Institute of Bioregulation, Kyushu University, Fukuoka, Japan; 8 Faculty of Chemistry, Biotechnology and Food Science, Norwegian University of Life Sciences (NMBU), 1432 Ås, Norway; 9 Institute for Medical Microbiology and Hospital Epidemiology, University of Marburg, 35037 Marburg, Germany

## Abstract

This most comprehensive analysis to date of γδ T cells in the murine uterus reveals them to compose a unique local T-cell compartment. Consistent with earlier reports, most cells expressed a canonical Vγ6Vδ1 TCR, and produced interleukin (IL)-17A upon stimulation. Nonetheless, contrasting with earlier reports, uterine γδ T cells were not obviously intraepithelial, being more akin to sub-epithelial Vγ6Vδ1^+^ T cells at several other anatomical sites. By contrast to other tissues however, the uterine compartment also included non-Vγ6^+^, IFN-γ-producing cells; was strikingly enriched in young mice; expressed genes hitherto associated with the uterus, including the progesterone receptor; and did not require microbes for development and/or maintenance. This notwithstanding, γδ T-cell deficiency severely impaired resistance to reproductive tract infection by *Candida albicans*, associated with decreased responses of IL-17-dependent neutrophils. These findings emphasise tissue-specific complexities of different mucosal γδ cell compartments, and their evident importance in lymphoid stress-surveillance against barrier infection.

## Introduction

Many tissues harbour two categories of lymphocytes which are largely noncirculating.^1^ The first category includes tissue-resident memory T (T_RM_) cells that enter tissues following priming in lymphoid organs and compose reservoirs of cells responsive to local reinfection and/or tumour challenge.^1^ T_RM_ have been particularly well-studied in skin, lung, and reproductive tissues.^1^ The second category comprises cells that home to the target organ developmentally, without requiring lymphoid priming.^2^ In mice, such lymphocytes include large subsets of γδ T cells with restricted TCR repertoires that have been particularly well-studied in the gut and epidermis. Clear counterparts of these cells were recently identified in human gut.^3^

Murine TCRγδ^+^ dendritic epidermal T cells (DETC) and small intestinal intraepithelial lymphocytes (IEL) are defined by their respective expressions of the Vγ5 and Vγ7 gene segments, which are developmentally selected by epithelial butyrophilin-like (Btnl) molecules, *Skint1* and *Btnl1*, respectively.^3–5^ Selection is independent of microbial colonisation,^3,6^ and occurs within discrete developmental windows: prenatal for DETC,^7,8^ and postnatal day 17–35 for Vγ7^+^ cells.^3^ It induces strong responsiveness to innate stimuli,^9^ such as stress-induced ligands for the activating NK receptor, NKG2D,^10^ while suppressing *Rorc* and *Sox13* expression, thereby diminishing the cells’ potential to produce interleukin (IL)-17A, in favour of IFN-γ, TNF, IL-13, and granzymes that contribute to the cells’ cytolytic potentials.^11^ Seemingly reflective of these effector capabilities, γδ^+^ T cells are associated with limiting skin and intestinal carcinogenesis.^12,13^

While some properties of tissue-associated γδ T cells are shared across anatomical sites, others seem site-specific, as was recently considered for γδ T cells in the gingiva.^14^

Thus, it is clearly important to better characterise each tissue-associated γδ T-cell compartment, particularly in the cases of organs housing T_RM_. In this regard, we have focused on the murine female reproductive tract (FRT). A TCRγδ^+^ uterine IEL compartment was described many years ago, that was limited to use of a quasi-monomorphic Vγ6Vδ1 TCR.^15^ Interestingly, cells with the same TCR were described in the lung, tongue, gut lamina propria, and dermis,^16^ although those cells are predominantly sub-epithelial, with potentially unique relationships with specific tissues.^17^

Most commonly, mucosal Vγ6Vδ1^+^ cells have been considered to be microbe-dependent,^18,19^ and those cells populating the gut lamina propria only expanded into a prevalent subset following oral infection, e.g. with *Listeria monocytogenes.*^20^ Apparently consistent with this, mucosal Vγ6Vδ1^+^ cells were shown to provide anti-microbial protection, particularly against re-challenge, and this was primarily ascribed to IL-17A production.^20–23^ Likewise, so-called γδ17 cells have been strongly implicated in inflammatory immunopathologies, including psoriasis, neuroinflammation, and cancer,^24–26^ although some have also been ascribed amphiregulin-dependent contributions to tissue homoeostasis.^14^

The cells’ production of IL-17A is considered to reflect a lack of developmental selection events akin to those shaping IEL compartments. Indeed, Vγ6Vδ1^+^ thymocytes have been reported to apoptose upon TCR cross-linking,^27^ although there are counter-arguments in favour of the cells’ selection.^9,28^ Conceivably, the lack of endogenous selecting elements might be compensated for by the development of γδ17 cells being driven by the microbiome, perhaps related to the cells’ provision of anti-microbial protection.

In this study, a comprehensive analysis of murine uterine T lymphocytes has revealed a unique TCRγδ^+^ population. In contrast to earlier reports,^15^ uterine cells were largely stromal, evoking sub-epithelial γδ T cells in the dermis and gut. Like many of those cells, most uterine TCRγδ^+^ cells expressed a canonical Vγ6Vδ1 TCR and produced IL-17A upon stimulation. Nonetheless, the cells were heterogeneous in including a minor subset of non-Vγ6^+^ cells producing IFN-γ. Furthermore, uterine γδ cells phenocopied epidermal and intestinal IEL in that their development and/or maintenance was regulated by a distinct time window in early life, and was independent of microbes. This notwithstanding, γδ cells provided non-redundant protection against vaginal *Candida albicans* infection of adult mice.

## Results

### A developmentally regulated, intrastromal uterine γδ compartment

**Fig. 1:**
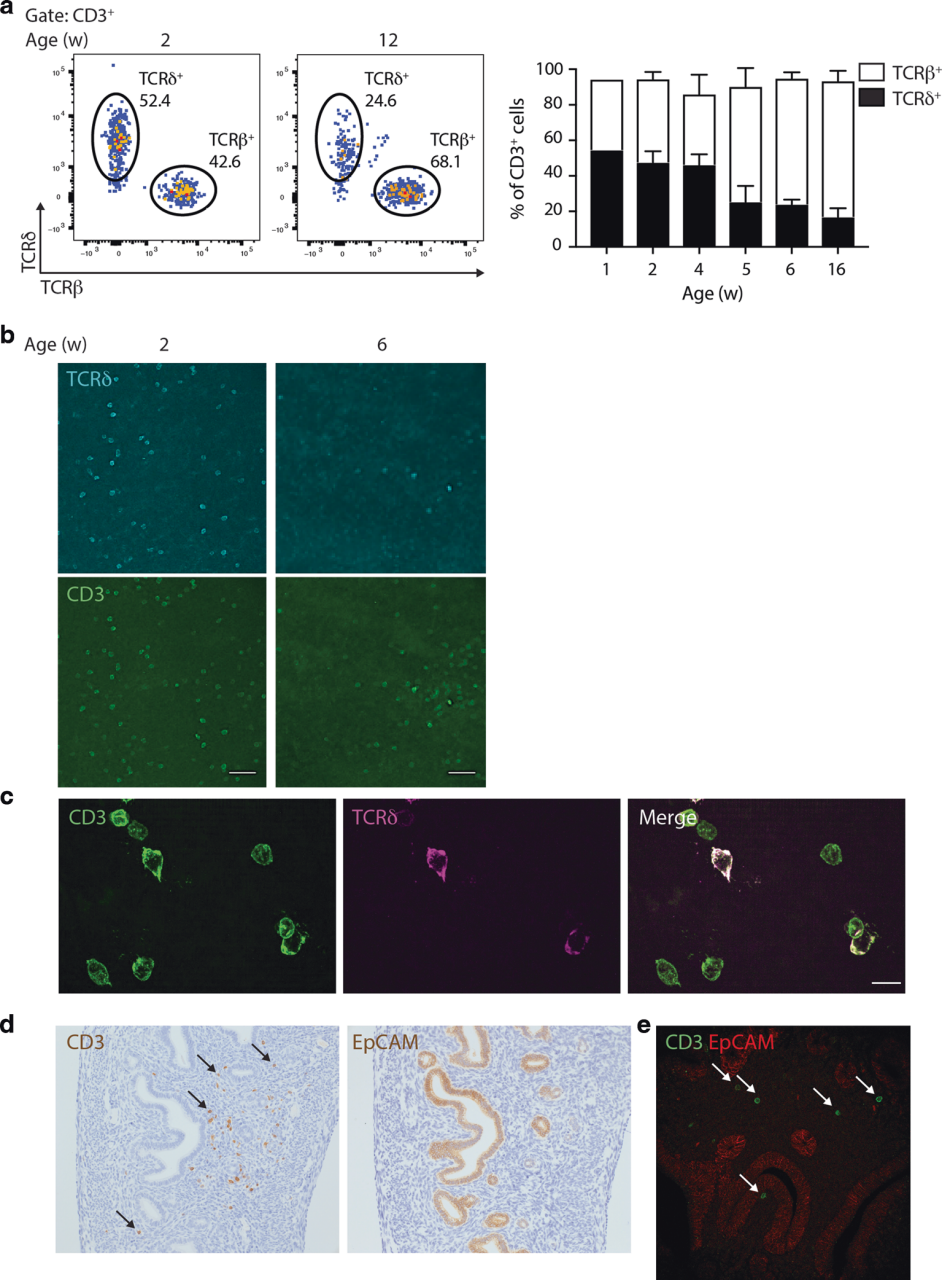
A major uterine γδ T-cell compartment, particularly in early life. **a** Left: Flow cytometry of CD3^+^ lymphocytes from the uterus of 2- and 12-week-old C57BL/6J mice. Representative plots are shown. Right: Uterine T-cell kinetics; the percentages of TCRδ^+^ and TCRβ^+^ cells (out of CD3^+^ cells) are indicated (*n* = 3–7 per condition; a pool of nine uteri is shown for 1-week-old mice). Data represent at least two independent experiments. Graph indicates mean ± SD. **b** CD3 and TCRδ staining were analysed by confocal microscopy on uterus whole-mounts of C57BL/6J mice of the indicated ages. Representative images from two independent experiments are shown (*n* = 3). Scale bars: 50 μm. **c** High-resolution images of CD3 and TCRγδ staining were obtained by iSIM on uterus whole-mounts of 2-week-old C57BL/6J mice (*n* = 3). Scale bars: 10 μm. CD3-TCRδ co-staining appears as purple-white. **d** CD3 and EpCAM staining were analysed by immunohistochemistry on FFPE uterine serial sections. Arrows indicate the position of several CD3^+^ cells. Representative images from four independent samples are shown. **e** CD3 and EpCAM staining were analysed by confocal microscopy on uterus FFPE sections. Representative images are shown.

By flow cytometry, TCRγδ^+^ cells accounted for over half the T cells in the uterus of mice aged 4 weeks old or younger (Fig. 1a; Supplementary Fig. 1a). Consistent with evidence that uterine γδ T-cell progenitors develop from late fetal thymi,^29^ γδ cells were already the predominant T-cell subtype by 1 week post-partum (Fig. 1a). However, unlike the case for DETCs, the representation of γδ T cells in the uterus overtly decreased in older mice, and by weeks 12–16 comprised <20% of T cells (Fig. 1a). This pattern did not reflect differential cell recovery, since it was also apparent when tissue whole-mounts were visualised by confocal microscopy (Fig. 1b). Visualisation in situ and flow cytometry analysis also showed that the decrease in γδ T-cell representation was one of absolute numbers as opposed to simply reflecting increasing numbers of αβ T cells (Fig. 1b; Supplementary Fig. 1b).

High-resolution instant structured illumination microscopy (iSIM) showed that the cells’ morphology was more lymphoid than dendritic, akin to intestinal IEL rather than DETC (Fig. 1c). Immunohistochemical staining of uterine serial sections and confocal microscopy showed that T cells were within the vicinity of epithelial EpCAM^+^ regions, but provided no evidence that T cells were intraepithelial (Fig. 1d, e). Indeed, confocal microscopy of uterus whole-mounts, together with quantitative 3D image analysis, showed that most TCRδ^+^ or CD3^+^TCRδ^−^ cells were separated by several cell-widths’ distance from EpCAM^+^ clusters, as opposed to being juxtaposed with them (Supplementary Fig. 1c, d).

### Limited TCR repertoire but functional heterogeneity

**Fig. 2:**
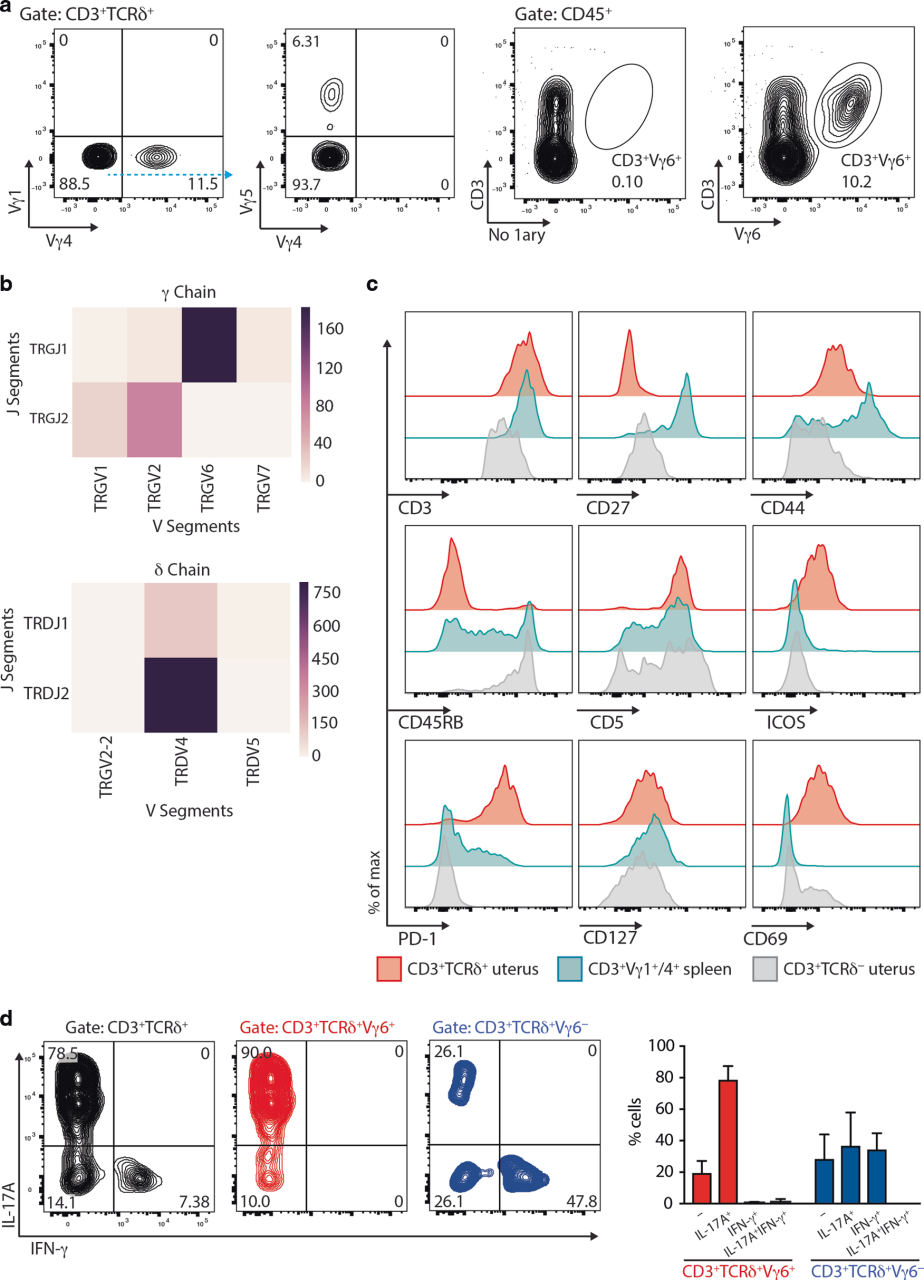
Surface phenotype, TCR usage, and functional properties of uterine γδ T cells. **a** Left: TCR usage of uterine γδ T cells was determined by flow cytometric analysis of Vγ1, Vγ4, and Vγ5 chains. Right: TCR usage of uterine γδ T cells determined by flow cytometry using the Vγ6-specific 1C10-1F7 antibody. Representative flow plots from three experiments are shown. **b** TCR deep-sequencing analysis of RNA from sorted uterine Vγ1^−^4^−^5^−^ cells (representative data from three biological replicates), showing the relative abundance of recombination events for gamma (top) and delta (bottom) gene segments. **c** The surface immunophenotype of uterine TCRγδ^+^ and CD3^+^TCRδ^−^ (i.e. TCRαβ^+^) T cells, and splenic Vγ1/4^+^ γδ T cells were determined by flow cytometry. Representative data from two experiments are shown. **d** Left: Uterine γδ T-cell suspensions were prepared and stimulated with PMA and ionomycin in the presence of Brefeldin A, with IL-17A and IFN-γ production assessed by intracellular staining and flow cytometric analysis in total (left), Vγ6^+^ (middle, red), and Vγ6^−^ (right, blue) γδ T cells. Right: Percentages of cytokine-secreting cells amongst Vγ6^+^ (red) and Vγ6^−^ (blue) cells were determined (*n* = 5 mice). Representative data from two experiments are shown. Graph indicates mean ± SD.

Next, γδ T cells isolated from the uterus of mice 2–3 weeks post-partum were assessed for TCR usage. A minor fraction stained with antibodies reactive to Vγ4, the TCR Vγ chain primarily used by lymphoid IL-17A-producing cells, but there was little staining with antibodies to Vγ1 (used by lymphoid IFN-γ-producing γδ cells) or Vγ5 (expressed by DETC) (Fig. 2a). Instead, most cells stained with a recently reported antibody specific for murine Vγ6^30^ (Fig. 2a; Supplementary Fig. 2a).

Moreover, by TCR deep sequencing of FACS-sorted TCRVγ1^−^4^−^5^−^ cells, ~80% of productive rearrangements corresponded to the canonical rearrangement of Vγ6 to Jγ1^15^ (Fig. 2b; Supplementary Table 1a). About 10% of productive rearrangements were of Vγ1 or Vγ2 rearrangements to Jγ2, which may reflect a mixture of non-isotypic allelic exclusion^31^ and/or αβ T cells in which Vγ2 rearrangements can be found. Also consistent with earlier studies, >80% of productive TCRδ rearrangements in TCRVγ1^−^4^−^5^−^ cells were canonical rearrangements of TRDV4 (which encodes TCRVδ1) to Jδ2.^15^ In addition, there were some productive Vδ1 rearrangements to Jδ1, such that all in all, Vδ1 rearrangements accounted for ~99% of productive sequences (Fig. 2b; Supplementary Table 1b).

The uterine γδ T-cell phenotype from mice aged 2–3 weeks (Fig. 2c, red) was then compared with uterine αβ T cells (Fig. 2c, grey) and TCRVγ1^+^ and Vγ4^+^ splenocytes which together comprise primary subtypes of lymphoid γδ^+^ T cells (Fig. 2c, blue). Unlike their comparator populations, the uterine TCRγδ^+^ T cells were mostly homogeneous, displaying a canonical surface phenotype: CD3^hi^ (consistent with Fig. 1b, above), CD27^−^, CD44^+^, CD45RB^−^, CD5^+^, ICOS^+^, CD127^lo^, CD69^+^, and PD-1^+^. Some of those traits also distinguished uterine TCRγδ^+^ cells from intestinal TCRγδ^+^ IEL and DETC, which are mostly CD45RB^+^ and CD5^(−)3^. The cells’ expression of ICOS, PD-1, and CD69 evoked several types of tissue-resident populations, including TCRαβ^+^ T_RM_ cells.^1^ Conversely, the relatively modest expression of CD127, which encodes the IL7Rα chain, contrasted with its high expression on other γδ17 cells.^32^

Although the expression of most markers was largely unimodal, consistent with the predominant expression of a single Vγ6Vδ1 TCR, there was some heterogeneity (e.g. note some CD45RB^+^ cells in left panel, middle row; Fig. 2c), which was further reflected in functional heterogeneity: thus, when stimulated with PMA + ionomycin, ~80% of cells produced IL-17A, whereas ~8% of cells produced IFN-γ but no IL-17A (Fig. 2d; Supplementary Fig. 2b). When phenotyped, the latter cells were mostly CD45RB^+^ and were all Vγ6^−^CD44^lo^, whereas IL-17A-producers were predominantly Vγ6^+^ and all CD45RB^−^CD44^+^ (Fig. 2d; Supplementary Fig. 2b). While these data are consistent with developmental pre-programming of CD45RB^−^CD44^+^ thymocytes toward IL-17A and of CD45RB^+^CD44^−^ thymocytes toward IFN-γ,^27^ they demonstrate greater heterogeneity than had been implied for the uterine compartment by earlier studies.^33^

### Tissue-specific Vγ6^+^ T cells

**Fig. 3:**
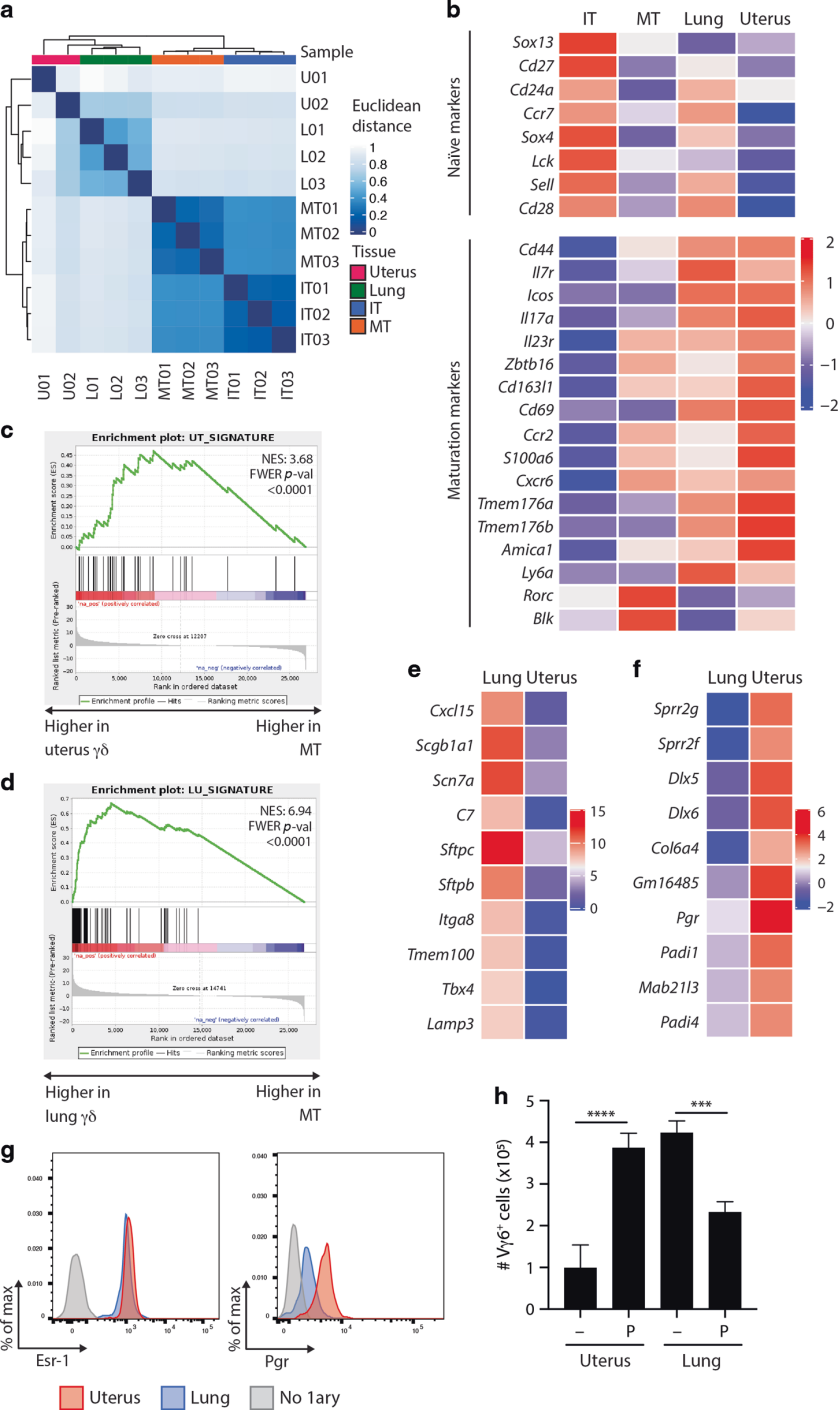
Uterine and pulmonary γδ T cells adapt to sites of residence. **a** Immature Vγ1^−^4^−^5^−^ thymocytes (IT, CD24^+^CD44^−^), mature Vγ1^−^4^−^5^−^ thymocytes (MT, CD24^−^CD44^+^), and pulmonary (L) and uterine (U) Vγ1^−^4^−^5^−^ γδ T cells were sorted and gene expression determined via RNAseq (2–3 independent samples per condition). A Euclidean clustered distance matrix heatmap was generated using overall gene expression. Normalisation and variance-stabilising transformation (VST) were applied on raw counts before plotting. **b** Heat map of immaturity and maturation-associated marker expression across the RNAseq datasets. Changes in transcript abundance between conditions are shown with *Z*-scores computed on the mean of the samples variance-stabilising transformation (VST). **c** Gene set enrichment analysis (GSEA) for uterus signature genes was performed for differentially expressed genes between mature Vγ1^−^4^−^5^−^ thymocytes and uterine Vγ1^−^4^−^5^−^ γδ T cells. The enrichment score (NES) and *p* value are reported. Genes were ranked based on the Wald statistic resulting from the differential expression analysis. **d** Gene set enrichment analysis (GSEA) for lung signature genes was performed for differentially expressed genes between mature Vγ1^−^4^−^5^−^ thymocytes and pulmonary Vγ1^−^4^−^5^−^γδ T cells. The enrichment score (NES) and *p* value are reported. Genes were ranked based on the Wald statistic resulting from the differential expression analysis. **e** Expression of the ten lung-specific genes most differentially expressed between uterine and pulmonary Vγ1^−^4^−^5^−^ γδ T cells. Changes in transcript abundance between conditions are shown with *Z*-scores computed on the mean of the samples variance-stabilising transformation (VST). **f** Expression of the top ten uterus-specific genes most differentially expressed between uterine and pulmonary Vγ1^−^4^−^5^−^ γδ T cells. Changes in transcript abundance between conditions are shown with *Z*-scores computed on the mean of the samples variance-stabilising transformation (VST). **g** Oestrogen receptor 1 (Esr-1) and progesterone receptor (Pgr) protein expression by lung (blue) and uterus (red) γδ T cells were determined by flow cytometry (*n* = 3). A negative control stained in the absence of primary antibody is shown in grey. Representative data from two experiments are shown. **h** Total numbers of Vγ6^+^ cells recovered from uterine or lung cell cultures following in vitro expansion in media optimised for γδ17 cells. When indicated, cultures were supplemented with progesterone (P) (*n* = 3). Graph indicates mean ± SD. Statistical significance was assessed by one-way ANOVA with Sidak’s multiple comparisons post-hoc test. ns not significant, ***p < 0.001, ****p < 0.0001.

Given that the uterine γδ T-cell compartment appeared unique, we sought a more detailed analysis of how uterine γδ T cells relate to γδ T-cell progenitors and to their counterparts in another mucosal site. Thus, we purified CD24^+^ immature Vγ1,4,5^−^ TCRγδ^+^ thymocytes (IT01, 02, 03); CD44^+^ mature Vγ1,4,5^−^ TCRγδ^+^ thymocytes (MT01, 02, 03); and Vγ1,4,5^−^ cells from the uterus (U01, 02) and from lungs (L01, 02, 03) (Supplementary Fig. 3), and subjected each to RNASeq. We used overall gene expression profiles to establish a distance clustering matrix (Fig. 3a). The immature thymocytes clustered together, with commonalities evident from blue-coloured squares for all nine possible comparisons. By contrast, there were fewer commonalities of IT samples with mature thymocytes, evident from comparing IT01/02/03 with MT01/02/03 (Fig. 3a). Mature thymocytes clustered together, but displayed little in common with lung samples, which also clustered together (Fig. 3a). One of the uterine samples, U02, showed some commonality with the three lung samples, whereas the other, U01, was more distantly related, consistent with the cells’ uniqueness.

In sum, we could conclude that lung and uterine Vγ1,4,5^−^ cells had differentiated beyond the status of mature thymocytes, displaying an enrichment in maturation-associated genes (Fig. 3b, lower panel) and a downregulation of markers of immature cells relative to Vγ1,4,5^−^ TCRγδ^+^ thymocytes^17^ (Fig. 3b, upper panel). Moreover, uterine and lung Vγ1,4,5^−^ cells were clearly not equivalent. To investigate the basis of this, we extracted tissue-specific gene enrichment signatures from across 17 different organs, wherein genes were deemed tissue-specific when their expression level was greater than or equal to fourfold higher relative to all other tissues analysed.^34^ We subsequently used gene set enrichment analysis (GSEA) to interrogate the representation of each tissue-specific signature in uterine and lung γδ cells, respectively, relative to mature Vγ6^+^ thymocytes, as shown in Fig. 3c, d and Supplementary Tables 2 and 3.

Of note, uterine γδ T cells displayed a high enrichment score and significant *p* value for the uterus signature, whereas lung γδ T cells displayed the highest enrichment score and lowest *p* value for the lung signature, as reflected in the graphs in Fig. 3c, d, wherein black bars denote the positions of specific genes from the uterus or lung-specific signatures relative to the differential expression of mature CD44^+^ γδ thymocytes versus γδ T cells from uterus (Fig. 3c) or lung (Fig. 3d).

The γδ T-cell expression of signature, tissue-associated genes was overt for lung γδ T cells and included genes encoding surfactant proteins (*Sftpb*, *Sftpc*) and those regulating surfactant protein maturation (*Lamp3*) (Fig. 3e). This was somewhat less overt for uterine γδ T cells, but they did express genes encoding small proline rich repeat peptides and peptidyl arginine deaminases that regulate the structural integrity of surface epithelium (*Sprr2f, Sprr2g, Padi4*), and the gene, *Pgr*, encoding progesterone receptor (Fig. 3f). To preclude cell contamination as underpinning site-specific transcripts in γδ gene profiles, tissue-associated γδ T cells were assessed by flow cytometry, as illustrated for the oestrogen receptor (Esr-1) and progesterone receptor (Fig. 3g). Given the strong differential expression of the progesterone receptor between uterine and pulmonary γδ T cells, we evaluated the impact of progesterone addition on γδ T-cell expansion. Uterine, but not lung, Vγ6^+^ cells expanded fourfold in the presence of progesterone (Fig. 3h). Thus, Vγ6^+^ cells populating different tissues had diverged transcriptionally and functionally, partly reflecting adaptation to sites of residence. Notwithstanding those differences, uterine and lung γδ T cells also showed many similarities, for example in co-stimulator receptor expression (Supplementary Fig. 4).

### Microbes are dispensable for uterine γδ T cells

**Fig. 4:**
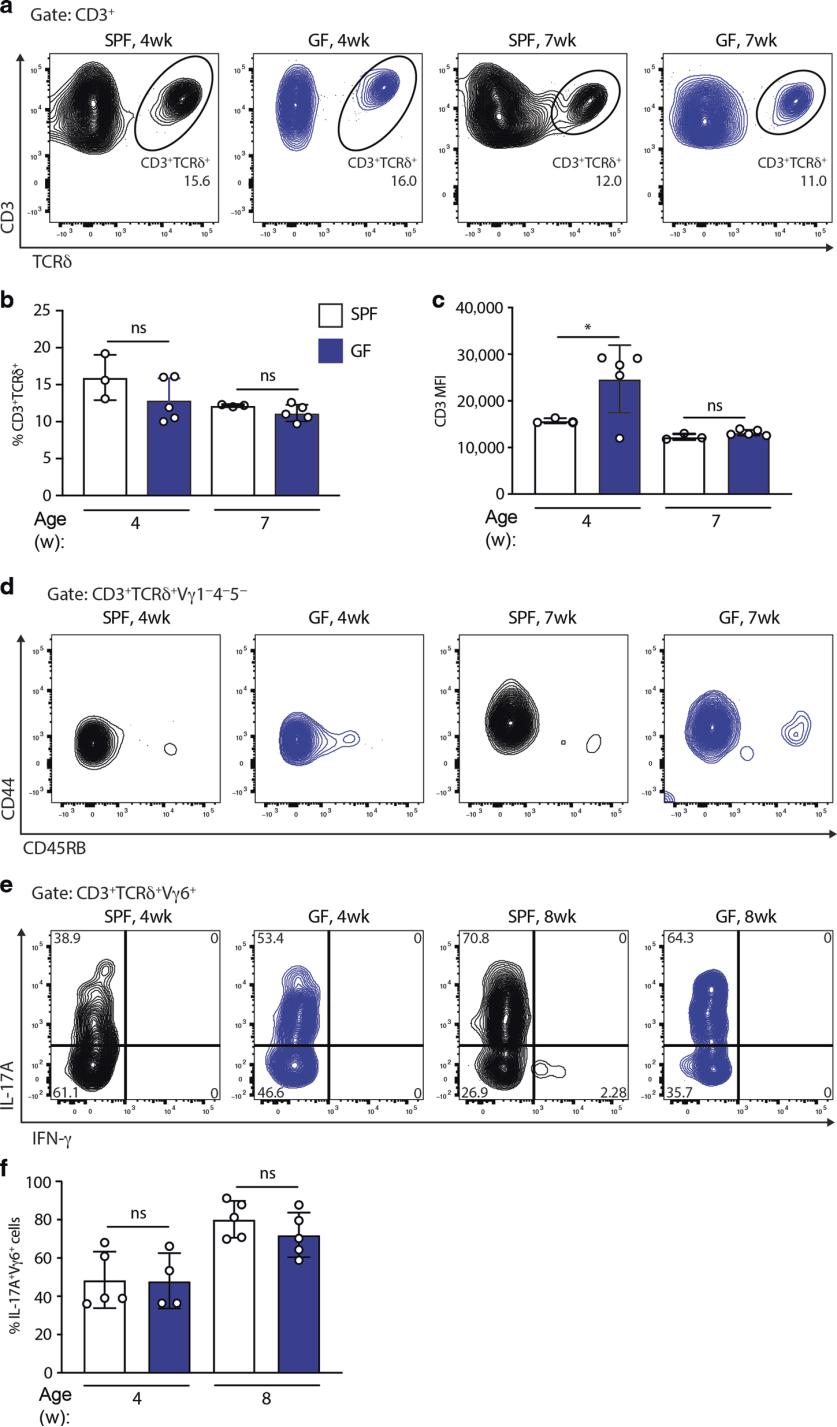
The microbiome is dispensable for uterine γδ T cells. **a** Uterine γδ T-cell staining (gated on CD3^+^ lymphocytes); **b** quantification; **c** CD3 expression; and **d** CD44 and CD45RB expression in SPF and germ-free (GF) C57BL/6J mice at 4 and 7 weeks (*n* = 3–5). Representative of four experiments. **e** Uterine γδ T-cell suspensions from SPF and GF mice were prepared and stimulated with PMA and ionomycin in the presence of Brefeldin A, with IL-17A and IFN-γ production assessed by intracellular staining and flow cytometric analysis in Vγ6^+^ γδ T cells. **f** Percentages of IL-17A-secreting cells amongst Vγ6^+^ cells were determined (*n* = 4–5 mice). Graph indicates mean ± SD. Statistical significance was assessed by one-way ANOVA with Sidak’s multiple comparisons post-hoc test. ns not significant, *p < 0.05.

Given the strong association of γδ17 cells with commensal and pathogenic bacteria,^18–20^ we next asked whether the cells required microbial colonisation for their development and/or maintenance. In fact, signature CD45RB^−^, CD44^+^ uterine γδ T cells displayed comparable frequencies, phenotypes and cytokine production profiles in 4-week-old and 7–8-week-old germ-free (GF) versus conventional specific pathogen-free (SPF) mice, albeit that the fluorescence intensity of CD3 was slightly increased in GF mice (Fig. 4a–f). Of note, absolute Vγ6^+^ cell counts were reduced in GF mice relative to their SPF counterparts (Supplementary Fig. 5a), which, together with overall reduced uterine size and cellularity (Supplementary Fig. 5b), may reflect a reduction in available niches for γδ T cells to populate.

**Fig. 5:**
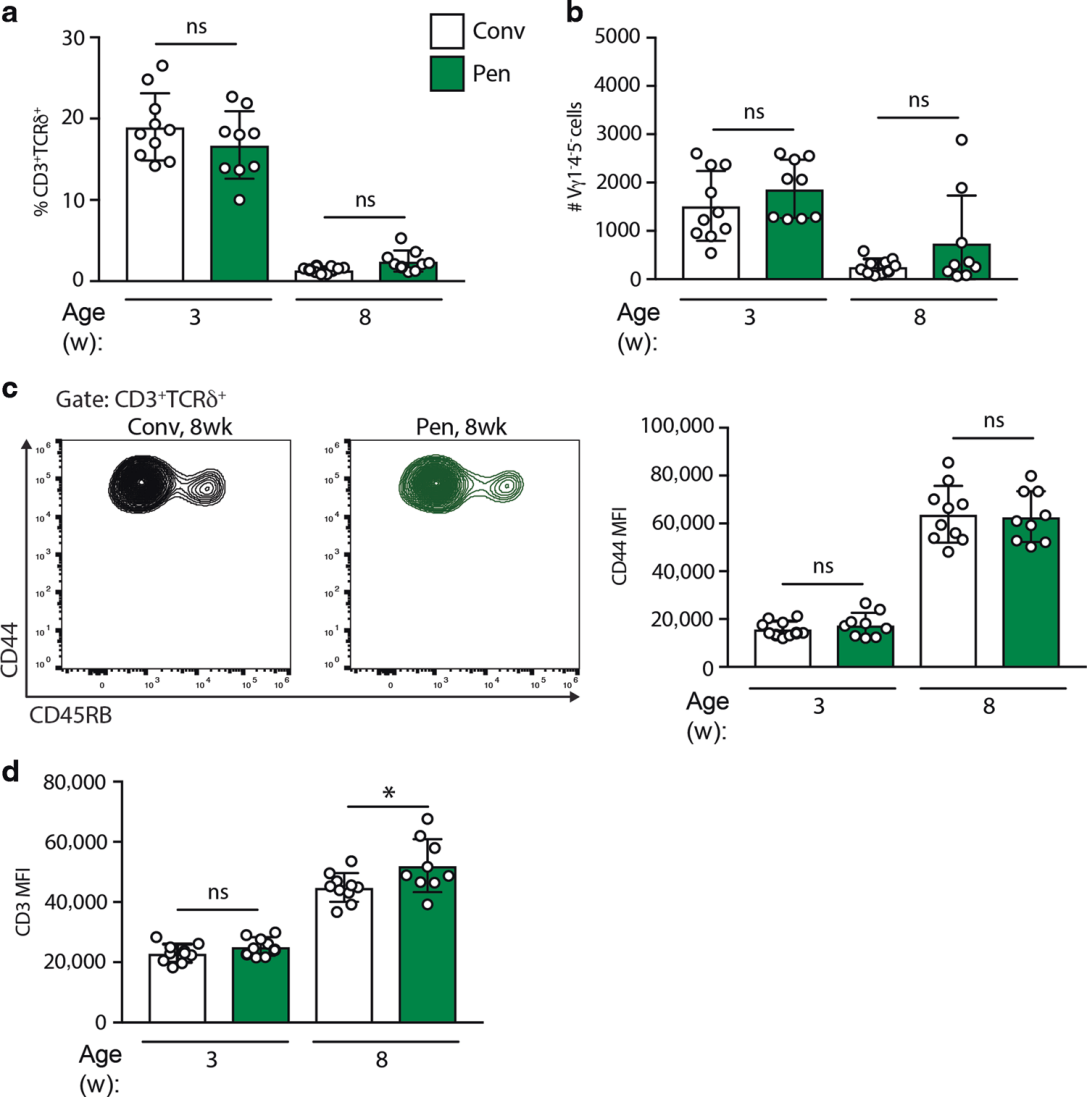
Environmentally enriched mice display comparable, age-dependent uterine γδ T cells. **a** Uterine γδ T-cell percentages; **b** absolute numbers; **c** CD44 and CD45RB expression; and **d** CD3 expression in conventional, pathogen-free (Conv), and microbially enriched (Pen) C57BL/6J mice at 3 and 8 weeks (*n* = 9–10). Graph indicates mean ± SD. Statistical significance was assessed by one-way ANOVA with Sidak’s multiple comparisons post-hoc test. ns not significant, *p < 0.05.

Although not dependent on microbes, we asked whether uterine γδ T cells might be expanded by environmental enrichment. Thus, we examined them in mice maintained in large pens supplemented with natural environmental materials, including woodchips, soil, and faecal content from farm animals, at the Norwegian University of Life Sciences. Females acclimatised to the pens were time-mated with males in cages and returned to pens following confirmation of pregnancy. Mice born from those litters were analysed at 3 and 8 weeks of age for their uterine γδ T-cell compartment. In fact, the numbers and representation of uterine cells were comparable with those housed under conventional, pathogen-free conditions, including a similar diminution between 3 and 8 weeks (Fig. 5a, b). Likewise, the cells’ signature phenotypes were comparable, albeit that the CD3/TCR fluorescence intensity was slightly increased in pen mice (Fig. 5b–d).

### γδ T-cell deficiency did not overtly affect breeding

**Fig. 6:**
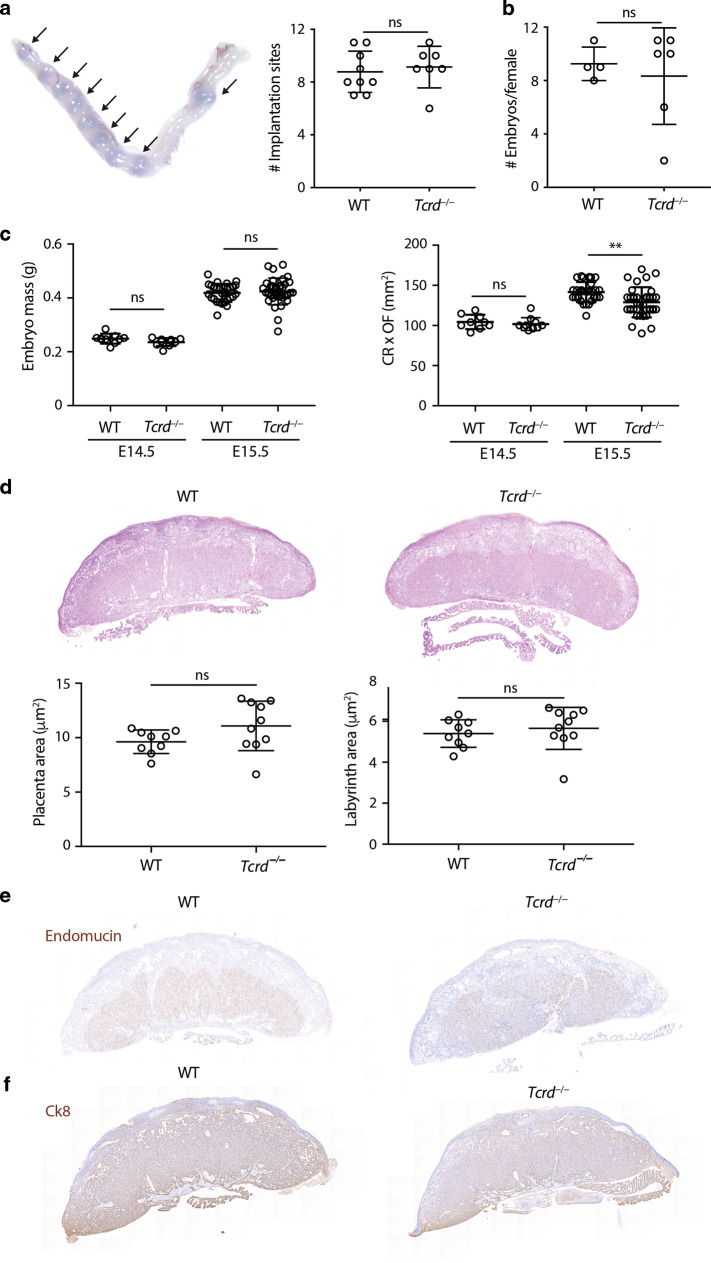
Pregnancies of γδ T-cell-deficient mice appear normal. WT or *Tcrd*^−/−^ females from the FVB background were time-mated to C57BL/6J males. **a** (Left) Implantation rates were analysed by i.v. injection of Evans blue at E5.5. A representative image of a gravid uterus, with arrows indicating implantation sites, is shown. (Right) Number of implantation sites per female (*n* = 7–9). **b** Total number of embryos per pregnant female at E14.5 and E15.5 (*n* = 4–6). **c** Embryo mass and size (CR crown-rump distance, OF occipito-frontal distance) were determined (E14.5: *n* = 9–10, E15.5: *n* = 28–34). **d** H&E staining of WT and *Tcrd*^−/−^ placentas and quantification of placental and labyrinth area (*n* = 9–10). **e** Endomucin immunohistochemistry of WT and *Tcrd*^−/−^ placentas. **f** Cytokeratin-8 (Ck8) immunohistochemistry of WT and *Tcrd*^−/−^ placentas. All graphs indicate mean ± SD. Statistical significance was assessed by unpaired *t*-test (**a**, **b**, **d**) or one-way ANOVA with Tukey’s multiple comparisons post-hoc test (**c**). ns not significant, **p < 0.01.

The large numbers of γδ T cells in the developing uterus led us to ask whether breeding or fecundity might be affected by γδ T-cell deficiency. Indeed, colony breeding analysis in our Institute suggested a potential effect, albeit very subtle, of γδ T-cell deficiency in long-term breeders (data not shown). We therefore interrogated breeding in γδ T-cell-deficient (*Tcrd*^−/−^) versus wild-type mice. Moreover, we examined whether any potential phenotypes might be exaggerated if mice were outbred, as would be the case in the wild. Thus, wild-type or *Tcrd*^−/−^ FVB females were time-mated to C57BL/6J males and implantation rates evaluated following intravenous delivery of Evans blue dye at E5.5 (Fig. 6a). The number of implantation sites per pregnant female was not significantly different between WT and *Tcrd*^−/−^ females (Fig. 6a).

In addition, pregnant females were analysed at E14.5 and E15.5 for embryo numbers, mass and size (crown-rump × occipito-frontal length; CR × OF)^35^ (Fig. 6b, c). This revealed comparable embryo numbers (Fig. 6b), and a very slight reduction in size (CR × OF) in pups from *Tcrd*^−/−^ females (Fig. 6c). While this might suggest some deficiency of *Tcrd*^−/−^ females to support optimal fetal development, CR × OF values across *Tcrd*^−/−^ and wild-type litters overlapped considerably, and histological analysis showed that placental microarchitecture was comparable (Fig. 6d). Moreover, there was comparable staining for endomucin, which marks placental endothelial cells, and for cytokeratin-8, which stains trophoblast cells^36^ (Fig. 6e, f).

### γδ T-cell deficiency increases susceptibility to *Candida albicans*

**Fig. 7:**
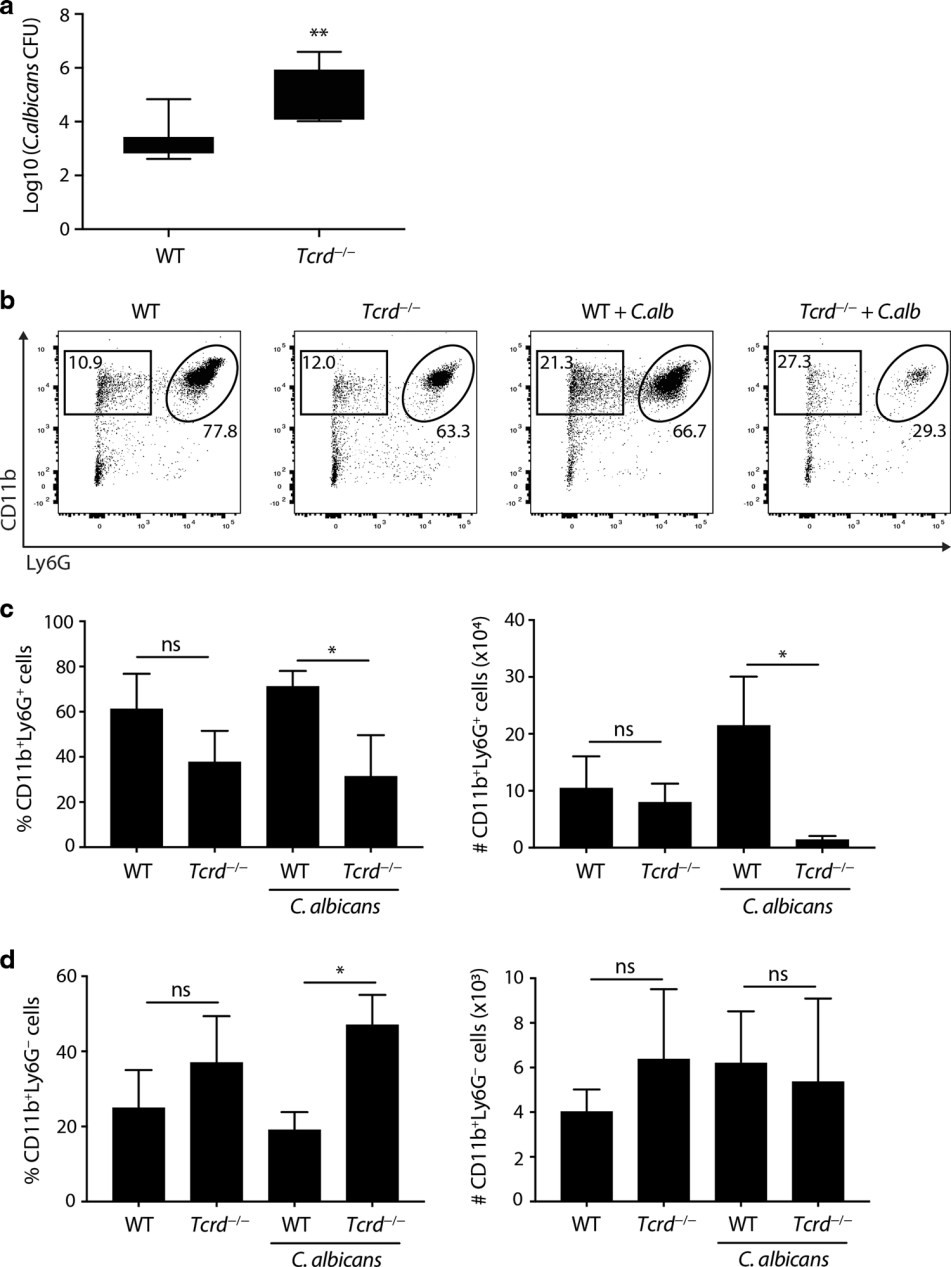
γδ T cells mediate protective responses to vaginal candidiasis. **a** C57BL/6J and *Tcrd*^−/−^ females (*n* = 5–8) were infected intravaginally with *C. albicans* 529 L and fungal burden assessed 7 days post infection in vaginal lavage and uterine lysate samples. The combined vaginal and uterine fungal burden is shown. Graph indicates mean ± SD. **b** Neutrophil (CD11b^+^Ly6G^+^) staining in vaginal cell suspensions was analysed by flow cytometry. **c** Neutrophil percentage and numbers in vaginal cell suspensions (*n* = 4–8). Graphs indicate mean ± SEM. **d** Percentage and numbers of CD11b^+^Ly6G^−^ cells in vaginal cell suspensions (*n* = 4–8). Graphs indicate mean ± SEM. Statistical significance was assessed by unpaired *t*-test (**a**) or one-way ANOVA with Sidak’s multiple comparisons post-hoc test (**c**, **d**). ns not significant *p < 0.05, **p < 0.01. Representative of five experiments.

Because uterine γδ T cells produce IL-17A which has been strongly associated with resistance to fungal infection in mice and in humans,^22,23,37^ we asked whether γδ T-cell deficiency might negatively impact host-protective responses to intravaginal infection by *C. albicans*. Indeed, *Tcrd*^−/−^ mice showed highly significant, two-log fold increases in fungal growth within the FRT (Fig. 7a). This correlated with greatly diminished infiltration of the tract by CD11b^+^Ly6G^+^ neutrophils, that are known to be regulated by IL-17A (Fig. 7b, c), whereas numbers of infiltrating CD11b^+^Ly6^lo^ monocytes were comparable, albeit that their percent representation was markedly increased because of the neutrophil deficiency (Fig. 7d). Thus, γδ T cells provide the reproductive tract with non-redundant protection against pathogenic fungi.

## Discussion

Ever since Stingl and Tigelaar showed independently that murine epidermal T cells primarily comprised γδ T cells,^38–40^ the association of specific γδ T-cell subsets with specific anatomical sites has been identified as a key signature of γδ T-cell biology. The significance of this has been reinforced by myriad observations, ranging from the conservation of tissue-associated γδ T-cell-like immunocytes in jawless vertebrates^41^ to increased skin cancer rates in mice lacking intraepidermal γδ T cells.^12^ These observations have collectively provoked the question as to how widespread are tissue-resident γδ T cells within the mouse and throughout evolutionary conservation. In that regard, early studies pointed to an intraepithelial γδ T-cell compartment in the uterus of adult mice, primarily expressing a canonical Vγ6Vδ1 TCR.^15^

Beyond confirming the existence of a uterine γδ T-cell compartment, the depth of this study revealed several unanticipated features. First, unlike skin or gut IEL, uterine γδ T cells are not juxtaposed with epithelial cells, but are intrastromal. Second, whereas uterine γδ T cells phenocopy other sub-epithelial compartments in being predominantly TCRVγ6Vδ1^+^ and biased toward IL-17A, there exists a discrete subpopulation of uterine IFN-γ-biased cells. And third, whereas uterine γδ T-cell compartments were found in all adult females examined, they were invariably diminished relative to those in young mice. In sum, the uterine γδ T-cell compartment is unique and more complex than hitherto reported.^15^

Tissue-associated γδ T-cell compartments are also known to express limited TCR repertoires, e.g. Vγ5Vδ1 in the epidermis, Vγ7Vδn in the murine gut, and Vγ4Vδn in the human colon.^3^ This has been attributed to the selection of cells bearing those TCRs by cognate Btnl molecules expressed by the local epithelium.^3–5^ Conversely, restricted TCR expression of sub-epithelial TCRγδ^+^ cells is not tissue-specific, with a canonical Vγ6Vδ1 rearrangement being dominant in the lung, dermis, lamina propria, and uterus.^16^ Whether or not this is a product of selection is a subject of ongoing examination.^9,11,28,42^

In most sites that Vγ6Vδ1 cells have been reported, γδ T cells have been shown to make pro-inflammatory host-protective responses, particularly to local infection by, for example, *Listeria monocytogenes* in the gut or *Bacillus subtilis* in the lung.^20,43^ Added to this, we now show that γδ T cells protect adult mice against *Candida* infection of the FRT.

In the cells’ absence, infiltrating neutrophil numbers were diminished, consistent with IL-17A production by uterine γδ T cells. As the effect of neutrophils in vulvovaginal candidiasis is controversial, with some reports highlighting a detrimental role,^44^ the association we highlight may reflect a strain-dependent protective axis of neutrophils and local γδ T cells. Moreover, γδ T cells may make additional non-redundant contributions to the protection of the FRT, e.g. via the promotion of tissue repair.^45–47^ Provocatively, uterine γδ T cells have been reported to fluctuate during the oestrus cycle,^48^ being highest in dioestrus when progesterone is highest. This might in part be explained by the direct proliferative/survival impact that we show progesterone to exert specifically on uterine γδ T cells. Whether γδ fluctuations may influence cyclical variations of other immune cells such as neutrophils, and thereby contribute to the establishment of the so-called window of vulnerability for FRT pathogens, has now to be considered.

In sum, our data provide additional evidence for the conclusion that γδ cells at various mucosal sites constitute a major, non-redundant line of defence to infection, albeit without any evidence to date of microbial antigen specificity.^49^ How such protection of mucosal surfaces is induced is unclear, but may be driven via the ‘adaptate’ biology of the TCR,^49^ and/or by innate receptors responding to molecular beacons of dysregulation, akin to DETC activation by NKG2D^10,50^ or γδ17 cell activation by IL-1 + IL-23:^51^ the so-called lymphoid stress-surveillance response.^2^

Notwithstanding the protective impacts of γδ T cells on *Candida*, uterine γδ T cells showed no dependence on microbes for their development or maturation. This was in contrast to microbial dependences cited for IL-17A-producing γδ T cells located in the dermis, intestinal lamina propria, and liver,^18,19^ but is in common with IEL, and with recently described meningeal Vγ6Vδ1 cells.^52^ Minor changes in CD3 expression were detected in GF and environmentally enriched mice relative to their SPF counterparts, which may reflect variations in the tissue inflammatory status in these mice.

Whichever factors drive the development and/or maintenance of uterine γδ T cells, they are evidently influenced by an ontogenetic time window, as reflected in the diminution of cell numbers with age. The preferential population of the uterus by γδ T cells in early life may reflect their early emergence from the embryonic thymus, whereas the cells’ diminution may reflect competition with other populations, e.g. T_RM_ cells, and/or age-associated changes in the uterine niche. While it is tempting to link this to sexual maturation, and despite the reported accumulation of γδ T cells at the maternal-fetal interface,^33,48,53^ none of our studies strongly implicated γδ T cells in reproductive fitness. However, this is not to exclude discrete roles for local γδ T cells in other aspects of reproductive biology, e.g. during post-partum uterine regression or secondary pregnancies, as may be revealed by future studies. Moreover, there is precedent for non-redundant properties of γδ T cells being germane uniquely to young mice.^54^

There have been increasing instances of myeloid and lymphoid cells expressing genes associated with their tissues of residence. This study expands this trend. A uterine transcriptional signature showed significant relatedness to the expression profile of uterine γδ T cells, which included functional expression of the progesterone receptor that was less well expressed by lung γδ T cells. Such findings emphasise that tissue-resident immune compartments should, at least partly, be viewed as an intrinsic component contributing to organ function in the same manner that we view epithelial and stromal cells. This contrasts with traditional perspectives by which tissue-associated immune cells infiltrate tissues in response to periodic challenge. Indeed, when one considers that Vγ6Vδ1 cells arise uniquely from the fetus, their developmental association with the uterus and other tissues evokes the biology of yolk sac-derived macrophages that contribute to organ function.^55^

One remaining issue is whether the human FRT harbours a major γδ T-cell compartment. This could be germane to the search for new pharmacological modalities to tackle increasing incidences of sexually transmitted infections. On the one hand, gut-resident γδ cells are conserved across rodents and primates, added to which human breast-associated and skin-associated γδ T cells have been identified.^56^ By contrast, it has been challenging to identify discrete compartments of human IL-17A-producing γδ T cells.^49^ While the reason for this difference is unknown, other differences between humans and mice may be connected, e.g. expression by humans of IL-8 which might substitute for IL-17 in activating neutrophils. Tackling such issues offers specific means by which to better understand human mucosal immunology.

## Methods

### Mice

SPF: C57BL/6J and *Tcrd*^−/−^ female mice were bred at the Francis Crick Institute.

GF: C57BL/6J mice were bred and maintained under axenic conditions at St. George’s, and at the University of Marburg.

Pen mice (Pen): A microbially enriched mouse housing model was designed at NMBU. Indoor pens built of galvanised steel (1.1 m × 2.4 m × 1.2 m) were prepared with woodchip bedding and enriched with soil, straw, and faecal content from farm animals. Three-week-old female and male C57BL/6JRJ mice were acclimatised for 1 week under conventional, pathogen-free conditions in individually ventilated cages (IVCs), before being distributed into different environments. Animals were primed in their respective housing environment for 4 weeks. For breeding, two pen-housed females and one male were brought together in IVCs enriched with pen material. After 10 days, the females returned to the pens to deliver. Female controls were mated under conventional, pathogen-free conditions in IVCs.

All experiments were performed according to the UK, German and Norwegian (FOTS-18012) animal protection laws.

### Flow cytometry

Uteri, vaginas, and lungs from mice were collected, minced, and digested using the Multi Tissue Dissociator kit-1 (Miltenyi). Samples were transferred into GentleMACS C tubes (Miltenyi) containing 2.5 mL digestion mix and incubated at 37 C for 40 min. Tissues were homogenised using GentleMACS program C and filtered through 70-μm strainers. Single-cell suspensions were stained with Live/Dead Aqua (Invitrogen), Fc-blocked (BD Biosciences) and stained with specific antibodies (TCRβ-BV605 (H57-597-BD Biosciences), TCRδ-BV421 (GL3-BioLegend), CD45-eV605 (30-F11-eBioscience), Vγ1-FITC (2.11-BioLegend), Vγ4-APC (UC3-10A6-BioLegend), Vγ5-PE (536, BioLegend), CD3-APCCy7 (17A2-BioLegend), CD27-FITC (LG.7F9-eBioscience), CD44-PECy7 (IM7-BioLegend), CD45RB-BV650 (16A-BD Biosciences), CD5-BV605 (UCHT2-BD Biosciences), ICOS-BV605 (7E.17G9-BD Biosciences), PD-1-BV605 (29F.1A12-BioLegend), CD127-BV711 (SB/199-BD Biosciences), CD69-PECy7 (H1.2F3-BioLegend), CD11b-FITC (M1/70-eBioscience), and Ly6G-PerCPCy5.5 (RB6-8C5-eBioscience). For identification of Vγ6^+^ cells, cell suspensions were incubated with anti-Vγ6 (1C10-1F7), followed by anti-mouse IgG1-APC (BioLegend).

For intracellular staining, cell suspensions were stimulated for 3 h with PMA 50 ng/mL, ionomycin 1 µg/mL, and brefeldin A 10 µg/mL. After surface staining, cells were treated with Foxp3-Fix/Perm buffer (BioLegend), followed by staining with anti-IL-17A-PE (TC11-18H10.1-BioLegend) and anti-IFN-γ-BV421 (XMG1.2-BioLegend). Alternatively, permeabilised cells were stained with anti-Pgr (Alpha PR6-Abcam) or anti-Esr (E115-Abcam), followed by goat-anti-mouse-IgG-PE (Thermo) or goat-anti-rabbit-F(ab′)_2_-AF647 (BioLegend). Cells were acquired with a BD X20 or Symphony and analysed with FlowJo (TreeStar).

### Microscopy

For whole mount staining, uteri were fixed in Zamboni, blocked with 5% BSA and stained with TCRδ-AF647 (Biolegend-118134), CD3-FITC (BD-553061), and EpCAM (Biolegend-118201), followed by anti-rat-AF568 (Invitrogen-A-11077). Z-Sections were acquired on a Leica SP5 microscope using a ×40 1.25 NA objective and processed and analysed with Fiji (NIH). To determine the position of γδ T cells and CD3^+^TCRδ^−^ cells relative to the epithelium, images were 3D rendered and the minimal distance between each TCRδ^+^ or CD3^+^TCRδ^−^ cell and EpCAM^+^ regions assessed via Definiens. For high-resolution analysis, samples were scanned using an Instant Structured Illumination Microscope (VT-iSIM, VisiTech International) using a ×100 1.45 NA objective and analysed with Nikon or Fiji (NIH).

Alternatively, mouse uteri were fixed in 10% NBF for 24 h at RT and paraffin-embedded. For immunohistochemistry, following antigen retrieval, peroxidase blocking, and incubation with 1% BSA, 3-μm sections were incubated with anti-CD3 (ab134096-Abcam) or EpCAM (14-5791-EBioscience). Antibodies were detected with goat-anti-rabbit-Biotin (BA-1000-Vector) or rabbit-anti-rat-Biotin (BA-4001-Vector), followed by detection with DAB (Vector). Slides were counterstained with hematoxilin, dehydrated, cleared, and mounted.

For confocal microscopy, slides were probed with anti-rabbit-AF647 and anti-rat-AF488, incubated with DAPI and mounted with Prolong Gold Antifade (Invitrogen).

### TCR sequencing

CD3^+^TCRδ^+^Vγ1^−^4^−^5^−^ cells were sorted from the uterus from C57BL/6J females into RLT Plus buffer. RNA was extracted with RNeasy micro kit Plus (Qiagen). TCR sequencing was performed with mouse Immunoverse TCRα/β/δ/γ kit (Archer Immunoverse).

### RNA sequencing

Uterus and lung CD3^+^TCRδ^+^Vγ1^−^4^−^5^−^ cells were sorted from C57BL/6J females, and thymic CD24^+^ (immature) or CD44^+^ (mature) CD3^+^TCRδ^+^Vγ1^−^4^−^5^−^ cells were sorted from E18.5 embryos, into RLT Plus buffer. RNA was extracted with RNeasy micro kit Plus (Qiagen). cDNA was prepared using the NuGEN Ovation RNA-Seq System, followed by library preparation with the NuGEN Ovation UltraLow system. Sequencing was carried out on a HiSeq-4000 (Illumina), with read lengths of 75 bp.

### Bioinformatic analysis

Raw reads were quality and adaptor trimmed using cutadapt-1.9.1. Reads were aligned and quantified using RSEM-1.3.0/STAR-2.5.2 against mouse genome GRCm38 and annotation 86 (Ensembl). Differential gene expression analysis was performed in R-3.6.0 using DESeq2 (version 1.24.0). Genes with an adjusted *p* value below 0.05 were deemed significant. Normalisation and variance-stabilising transformation (VST) were applied before performing euclidean distance-based clustering.

GSEA (version 3.0) was performed using Preranked analysis with the classic scoring scheme. Gene lists were generated with the results of differential expression analysis, and ranked using the Wald statistic. Uterus and lung genesets were generated with signatures from Supplementary Table 7 of ref. ^34^

Heatmaps were made in R-3.6.0 using ComplexHeatmap (version 2.0.0). VST was applied on raw counts. Then, for each experimental condition mean VST was computed across samples and used to compute *Z*-scores. For heatmaps of uterus and lung signatures, genes were ranked based on the differences mean(Uterus) − mean(Lung) and mean(Lung) − mean(Uterus), respectively.

### γδ17 expansion protocol

Uterine and lung γδ17 cells were expanded using a protocol adapted from McKenzie et al.^57^ Uterine or lung cell suspensions were cultured at a 1 × 10^6^ cells/mL in phenol red-free RPMI containing 10% charcoal-treated FCS, glutamine, Pen/Strep, 1 mM pyruvate, β-mercaptoethanol, and 1X NEAA (complete medium), supplemented with 5 ng/mL rIL-23 (eBioscience), 5 ng/mL rIL-1β (R&D Systems), 10 μg/mL anti-IFN-γ (eBioscience), and 1 μg/mL indomethacin (Sigma-Aldrich), for 3 days in 96-well plates coated with 1 μg/mL anti-TCRδ (eBioscience). Cells were harvested and re-plated for 3 days in the same medium, without TCR stimulation. Finally, cells were harvested and incubated for 3 days in complete medium with 20 ng/mL rIL-7 (Peprotech). Throughout the culture protocol, 1 μM progesterone (Sigma-Aldrich) or DMSO were added.

### Analysis of pregnancy

WT FVB or *Tcrd*^−/−^ females were timed mated with C57BL/6J males. For analysis of implantation, females were injected i.v. with 1% Evans Blue 5 days post coitum. Uteri were harvested after 3 min, and implantation sites detected as areas of Evans Blue accumulation. In addition, females were also culled at E14.5 or E15.5. Following uterus excision, embryos were enumerated, weighed and the CR and OF length measured. Placentas were harvested and fixed in NBF for histological analysis. FFPE placental sections were stained with H&E, and endomucin and cytokeratin-8 immunohistochemistry were performed.

### Vulvovaginal candidiasis

Three days prior to infection, 100 µg of estradiol-17-valerate (Sigma) in sesame oil were administered subcutaneously. For infection, mice were anesthetised and inoculated intravaginally with 5 × 10^6^ cfu of *C. albicans* 529 L in 10 µL of PBS. On day 7 post infection, mice were culled and lavaged with 100 µL of PBS for fungal burden assessment. Uterine lysates were homogenised in GentleMACS tubes, programme E. The burden was determined by plating serial dilutions of vaginal and uterine samples in YEPD + chloramphenicol plates.

### Statistical analysis

Differences between experimental groups were analysed using two-tailed Student’s *t* test. When multiple experimental groups were present, one-way ANOVA with Sidak’s post-hoc test was applied. To establish statistical significance of changes over time, we used one-way ANOVA with a post-hoc test for linear trend.

## Supplementary information

Supplementary Figure S1

Supplementary Figure S2

Supplementary Figure S3

Supplementary Figure S4

Supplementary Figure S5

Supplementary Tables
