# Supplementary figures and images for: γδ T cells compose a developmentally regulated intrauterine population and protect against vaginal candidiasis
Source: Mucosal Immunol. 2020 May 29;13(6):969–81. doi: 10.1038/s41385-020-0305-7 (PMC7567646; doi:10.1038/s41385-020-0305-7)

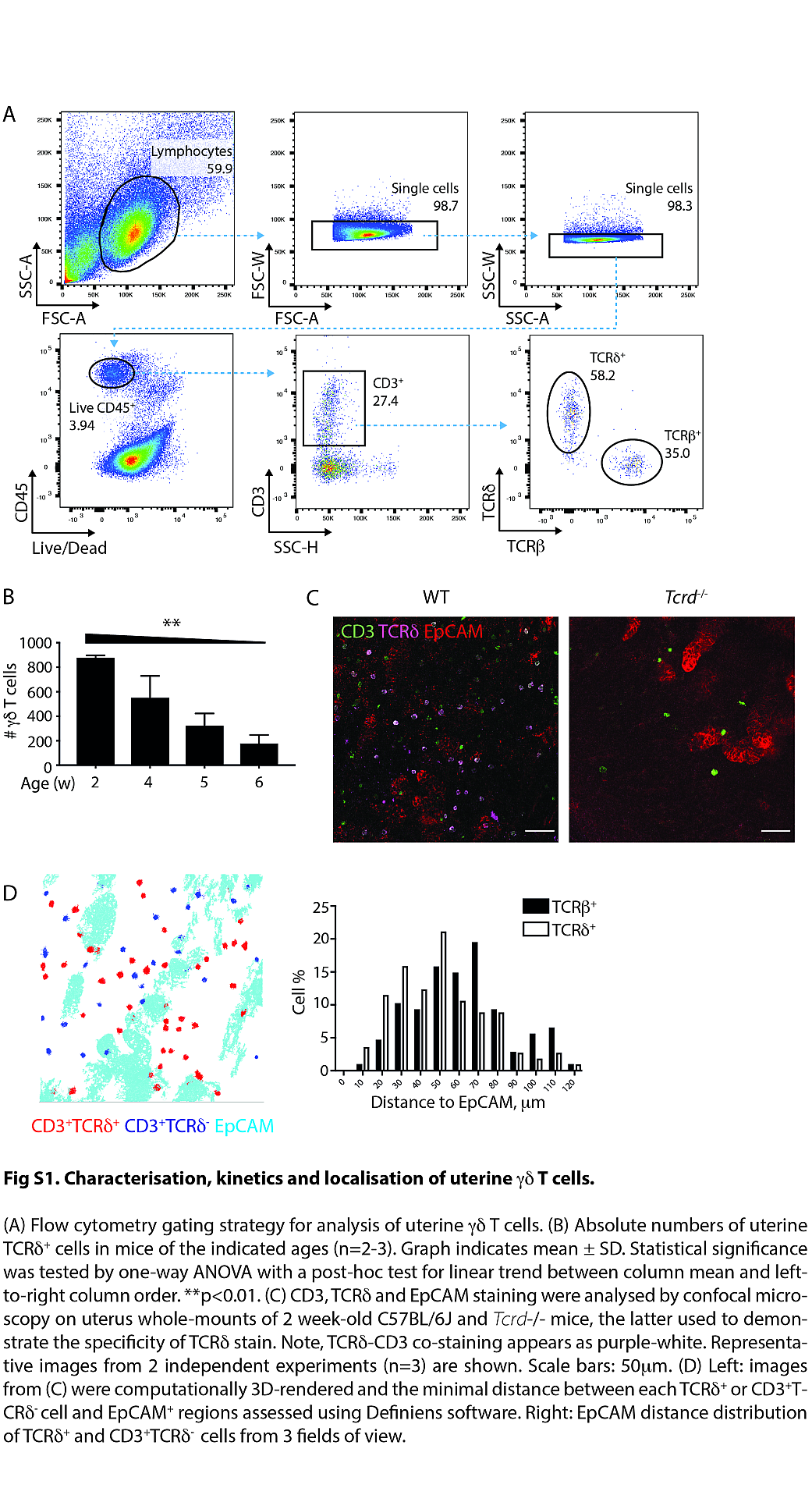

Supplement: Supplementary file 1 — Supplementary Figure S1 [file 41385_2020_305_MOESM1_ESM.tif]

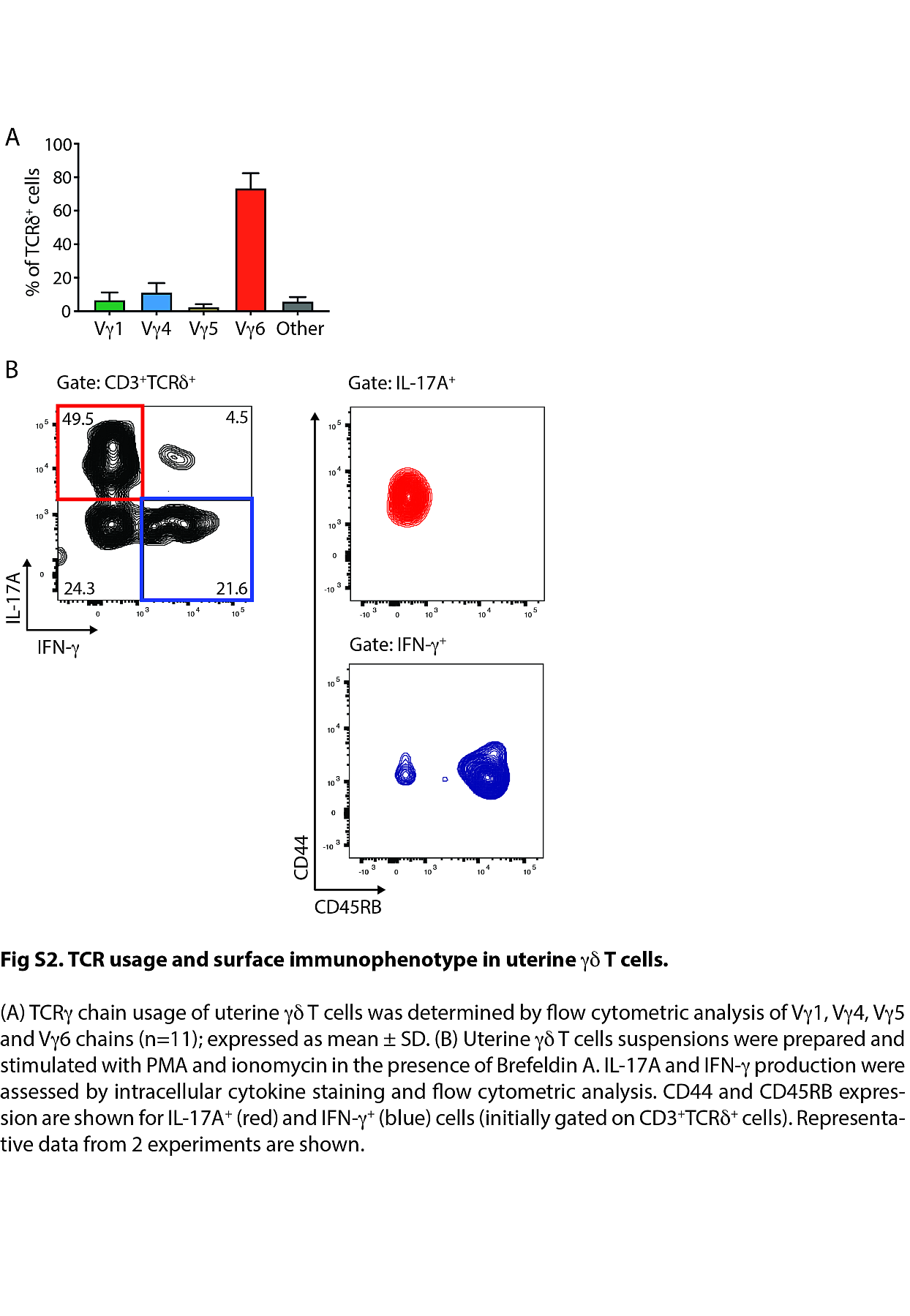

Supplement: Supplementary file 2 — Supplementary Figure S2 [file 41385_2020_305_MOESM2_ESM.tif]

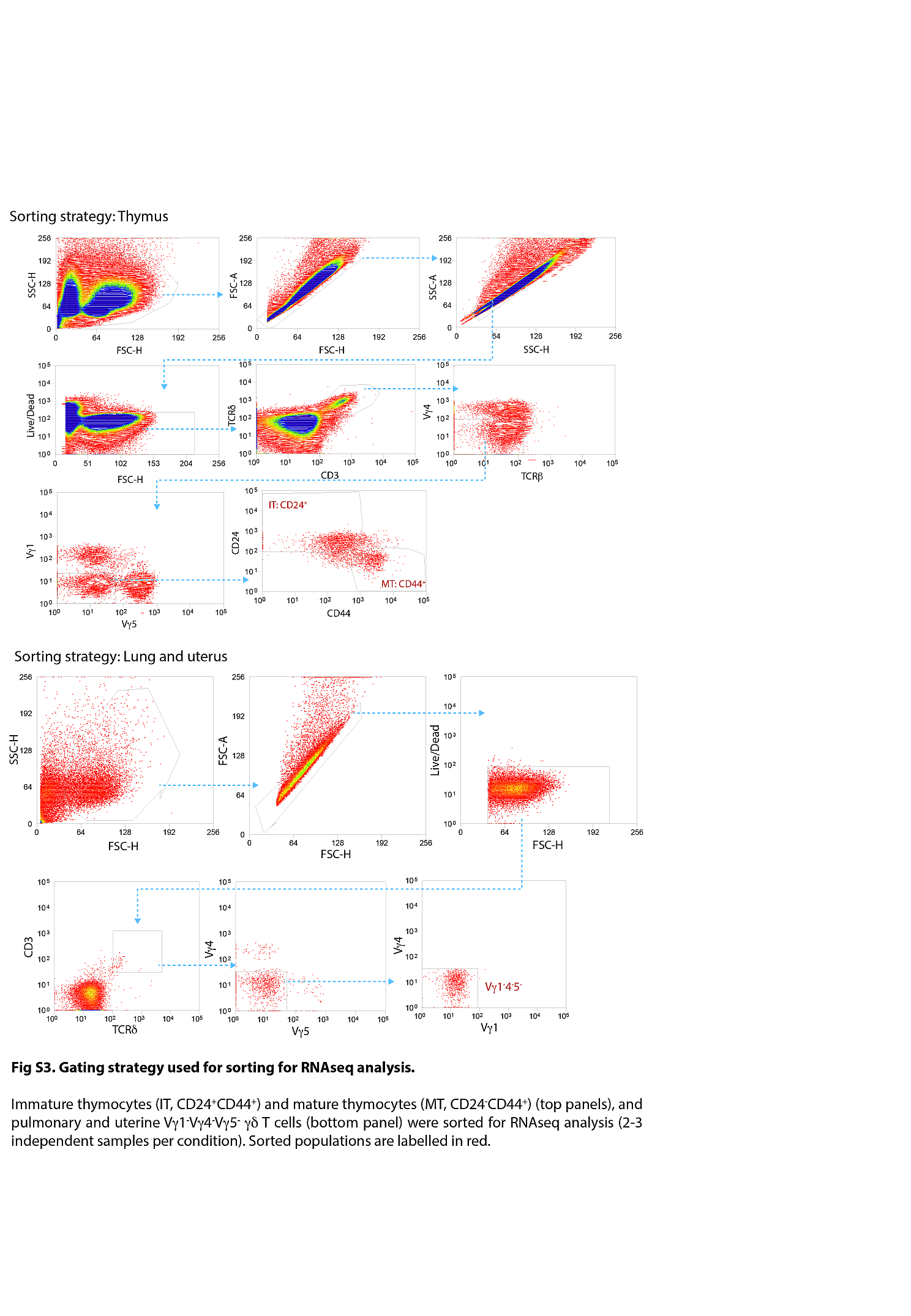

Supplement: Supplementary file 3 — Supplementary Figure S3 [file 41385_2020_305_MOESM3_ESM.tif]

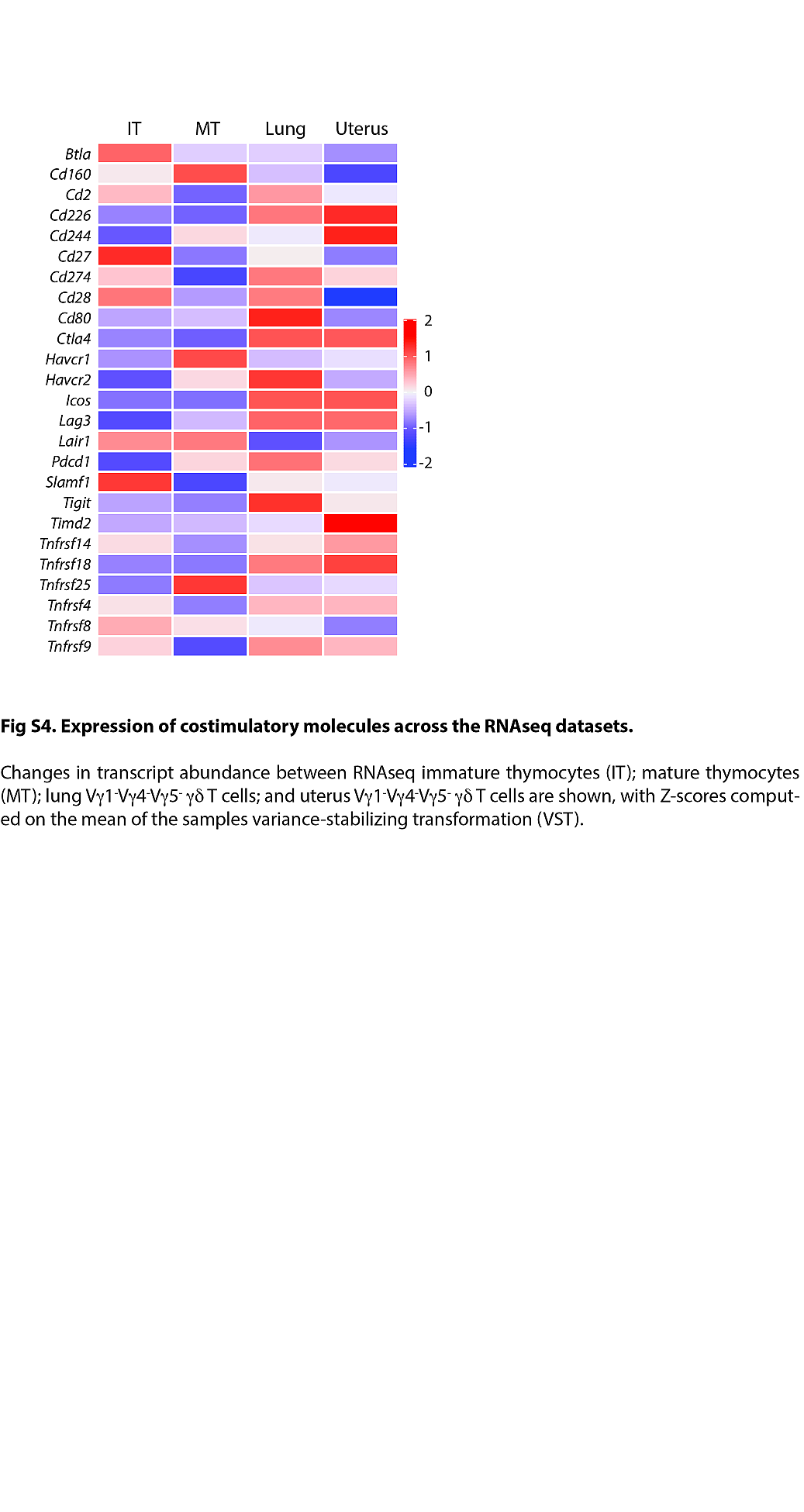

Supplement: Supplementary file 4 — Supplementary Figure S4 [file 41385_2020_305_MOESM4_ESM.tif]

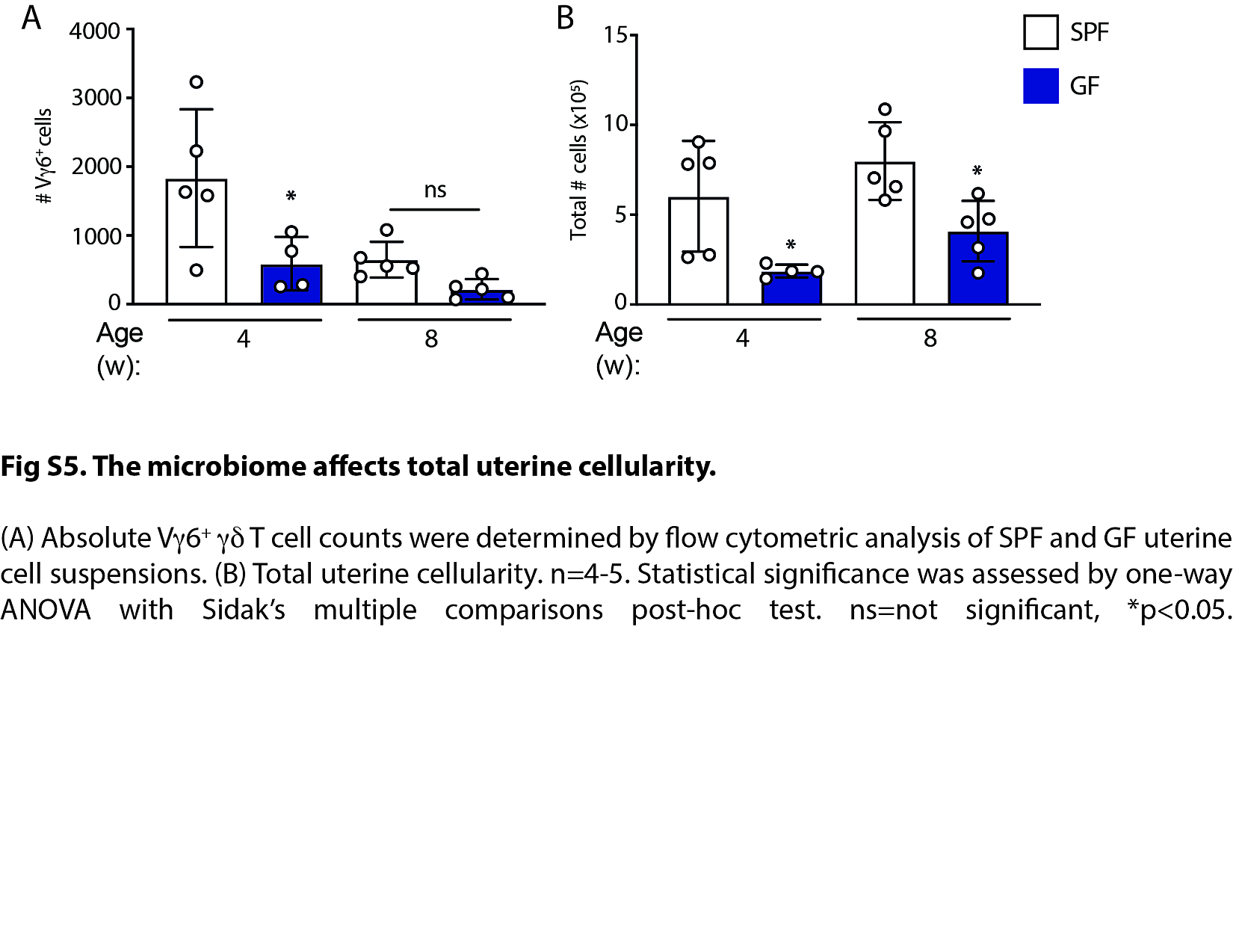

Supplement: Supplementary file 5 — Supplementary Figure S5 [file 41385_2020_305_MOESM5_ESM.tif]
