# Supplementary material for: γδ T cells compose a developmentally regulated intrauterine population and protect against vaginal candidiasis
Source: Mucosal Immunol. 2020 May 29;13(6):969–81. doi: 10.1038/s41385-020-0305-7 (PMC7567646; doi:10.1038/s41385-020-0305-7)
Supplement: Supplementary file 6 — Supplementary Tables [file 41385_2020_305_MOESM6_ESM.doc]

**Supplementary Table 1**

A. Productive γ chain CDR3 rearrangements in uterine Vγ1-4-5- cells.

| **CDR3 Sequence** | **Predicted V Region** | **CDR3 Translation** | **Freq** |
| --- | --- | --- | --- |
| TGTGCATGCTGGGATAGCTCAGGTTTTCACAAGGTATTT | TRGV6 | CACWDSSGFHKVF | 78.97 |
| TGTGCAGTCTGGTATAGCTCGGGCTTTCACAAGGTATTT | TRGV1 | CAVWYSSGFHKVF | 9.01 |
| TGTGCAGTATATAGCTCGGGCTTTCACAAGGTATTT | TRGV2 | CAVYSSGFHKVF | 7.73 |
| TGTGCCTCCTGGGCTGGATATAGCTCAGGTTTTCACAAGGTATTT | TRGV7 | CASWAGYSSGFHKVF | 2.58 |
| TGTGCATTCTTGGATAGCTCAGGTTTTCACAAGGTATTT | TRGV6 | CAFLDSSGFHKVF | 0.43 |
| TGTGCAGTCTGGTATAGCTCGGGCTTTCTCAAGGTATTT | TRGV1 | CAVWYSSGFLKVF | 0.43 |
| TGTGCAGTATATAGCTCGGGCTTTCACAAAGTATTT | TRGV2 | CAVYSSGFHKVF | 0.43 |

Canonical TRGV6 CDR3 is shown in red.

B. Top 10 productive  chain CDR3 rearrangements in uterine Vγ1-4-5^-^ cells.

| **CDR3 Sequence** | **Predicted V Region** | **CDR3 Translation** | **Freq** |
| --- | --- | --- | --- |
| TGTGGGTCAGATATCGGAGGGAGCTCCTGGGACACCCGACAGATGTTTTTT | TRDV4 | CGSDIGGSSWDTRQMFF | 84.30 |
| TGTGGGTCAGATATCGGAGGGATACGAGCTACCGACAAACTCGTCTTT | TRDV4 | CGSDIGGIRATDKLVF | 6.46 |
| TGTGGGTCAGATATCGGAGGGATACGAGCCGACAAACTCGTCTTT | TRDV4 | CGSDIGGIRADKLVF | 2.90 |
| TGTGGGTCAGATATCGGAGGGACCGACAAACTCGTCTTT | TRDV4 | CGSDIGGTDKLVF | 2.64 |
| TGTGCCTCGGATCGGAGGGATACGACCGACAAACTCGTCTTT | TRDV5 | CASDRRDTTDKLVF | 1.19 |
| TGTGGGTCAGATAGGGATACGAGCACCGACAAACTCGTCTTT | TRDV4 | CGSDRDTSTDKLVF | 0.79 |
| TGTGGGTCAGATATCGGAGGGAGCTCCTGGACACCCGACAGATGTTTTTTT | TRDV4 | CGSDIGGSSWTPDRCFF | 0.53 |
| TGTGGGTCAGATAGGGATACGAGCACCGTCAAACTCGTCTTT | TRDV4 | CGSDRDTSTVKLVF | 0.40 |
| TGTGTTTCAGATATCGGAGGGAGCTCCTTTGACACCCGACCTATTTTTTTT | TRDV4 | CVSDIGGSSFDTRPIFF | 0.13 |
| TGTGGGTCAGATATCGGAGGGAGCTCCTGGGCACCCGACAGATGTTTTTTT | TRDV4 | CGSDIGGSSWAPDRCFF | 0.13 |
| TGTGGGTCAGATATCGGAGGGAGCTCCTGGACACTCGACAGATGTTTTTTT | TRDV4 | CGSDIGGSSWTLDRCFF | 0.13 |
| TGTGGGTCAGATCGGAGGGATACGTACCGACAAACTCGTCTT | TRDV4 | CGSDRRDTYRQTRL | 0.13 |
| TGTGGGTCAGATCGGAGGGATACGTACGACAAACTCGTCTTT | TRDV4 | CGSDRRDTYDKLVF | 0.13 |
| TGTGGGTCAGATAGGGATACGAGCACCAACAAACTCGTCTTT | TRDV4 | CGSDRDTSTNKLVF | 0.13 |

Canonical TRGV4 (Vδ1) CDR3 is shown in red.

**Supplementary Table 2**

**Ranking of tissue-specific gene signatures enriched in uterine γδ T cells.**

| NAME | Signature size | ES | NES | NOM p-val | FDR q-val | FWER p-val | RANK AT MAX | LEADING EDGE |
| --- | --- | --- | --- | --- | --- | --- | --- | --- |
| BR | 480 | 0.179 | 4.614 | 0 | 0 | 0 | 13417 | tags=70%, list=53%, signal=145% |
| UT | 38 | 0.501 | 3.684 | 0 | 0 | 0 | 7364 | tags=79%, list=29%, signal=111% |
| LU | 78 | 0.292 | 3.052 | 0 | 0 | 0 | 6644 | tags=55%, list=26%, signal=74% |
| FS | 86 | 0.200 | 2.172 | 0.004 | 0.006 | 0.015 | 13011 | tags=71%, list=51%, signal=144% |
| LI | 114 | 0.172 | 2.172 | 0 | 0.004 | 0.015 | 13302 | tags=69%, list=52%, signal=144% |
| ST | 19 | 0.346 | 1.823 | 0.016 | 0.028 | 0.104 | 12663 | tags=84%, list=50%, signal=167% |
| SIN | 61 | 0.174 | 1.617 | 0.036 | 0.073 | 0.285 | 13128 | tags=69%, list=51%, signal=142% |
| HE | 31 | 0.241 | 1.562 | 0.056 | 0.084 | 0.369 | 17712 | tags=94%, list=69%, signal=306% |
| KI | 91 | 0.136 | 1.535 | 0.058 | 0.087 | 0.415 | 17003 | tags=80%, list=67%, signal=240% |
| OV | 47 | 0.166 | 1.332 | 0.148 | 0.182 | 0.719 | 16940 | tags=83%, list=66%, signal=247% |
| VG | 12 | 0.246 | 1.046 | 0.369 | 0.453 | 0.978 | 17098 | tags=92%, list=67%, signal=278% |
| LIN | 20 | 0.190 | 1.029 | 0.400 | 0.437 | 0.983 | 13013 | tags=70%, list=51%, signal=143% |
| AG | 27 | 0.130 | 0.819 | 0.686 | 0.686 | 1 | 18409 | tags=85%, list=72%, signal=306% |

Gene set enrichment analysis (GSEA) for tissue-specific signature genes was performed for differentially expressed genes between mature thymic and uterine γδ T cells. BR: Brain, LI: Liver, KI: Kidney, AG: Adrenal gland, SP: Spleen, LU: Lung, HE: Heart, TH: Thymus, OV: Ovary, ST: Stomach, FS: Forestomach, SIN: Small intestine, LIN: Large intestine, UT: Uterus, VG: Vesicular gland. ES: Enrichment score, NES: Normalized enrichment score, NOM p-val: Nominal p-value, FDR: False discovery rate, FWER: Familywise-error rate.

**Supplementary Table 3**

**Ranking of tissue-specific gene signatures enriched in pulmonary  T cells.**

| NAME | Signature size | ES | NES | NOM p-val | FDR q-val | FWER p-val | RANK AT MAX | LEADING EDGE |
| --- | --- | --- | --- | --- | --- | --- | --- | --- |
| LU | 78 | 0.660 | 6.937 | 0 | 0 | 0 | 5133 | tags=86%, list=20%, signal=107% |
| BR | 480 | 0.215 | 5.355 | 0 | 0 | 0 | 14917 | tags=80%, list=58%, signal=188% |
| ST | 19 | 0.433 | 2.303 | 0 | 0.003 | 0.005 | 14472 | tags=100%, list=57%, signal=231% |
| UT | 38 | 0.310 | 2.282 | 0.002 | 0.002 | 0.005 | 9564 | tags=68%, list=37%, signal=109% |
| OV | 47 | 0.275 | 2.244 | 0.002 | 0.002 | 0.006 | 11994 | tags=74%, list=47%, signal=140% |
| LI | 114 | 0.170 | 2.148 | 0.002 | 0.004 | 0.014 | 12919 | tags=68%, list=51%, signal=136% |
| KI | 91 | 0.183 | 2.030 | 0.009 | 0.009 | 0.035 | 12162 | tags=66%, list=48%, signal=126% |
| SP | 152 | 0.133 | 1.935 | 0.010 | 0.014 | 0.061 | 17256 | tags=81%, list=68%, signal=249% |
| FS | 86 | 0.152 | 1.626 | 0.045 | 0.059 | 0.263 | 17773 | tags=85%, list=70%, signal=279% |
| HE | 31 | 0.240 | 1.593 | 0.047 | 0.064 | 0.308 | 11992 | tags=71%, list=47%, signal=134% |
| LIN | 20 | 0.230 | 1.206 | 0.241 | 0.312 | 0.867 | 12001 | tags=70%, list=47%, signal=132% |
| SIN | 61 | 0.125 | 1.160 | 0.269 | 0.337 | 0.908 | 14788 | tags=70%, list=58%, signal=167% |
| AG | 27 | 0.162 | 1.006 | 0.442 | 0.497 | 0.982 | 8161 | tags=48%, list=32%, signal=71% |
| MU | 75 | 0.075 | 0.761 | 0.781 | 0.823 | 1 | 12046 | tags=55%, list=47%, signal=103% |
| VG | 12 | 0.176 | 0.744 | 0.782 | 0.791 | 1 | 18892 | tags=92%, list=74%, signal=353% |

Gene set enrichment analysis (GSEA) for tissue-specific signature genes was performed for differentially expressed genes between mature thymic and pulmonary γδ T cells.
